# Childhood adversity, mental health, and oxidative stress: A pilot study

**DOI:** 10.1371/journal.pone.0215085

**Published:** 2019-04-26

**Authors:** Sarah R. Horn, Leslie D. Leve, Pat Levitt, Philip A. Fisher

**Affiliations:** 1 University of Oregon, Department of Psychology, University of Oregon, Eugene, OR, United States of America; 2 University of Oregon, College of Education, University of Oregon, Eugene, OR, United States of America; 3 University of Southern California, Keck School of Medicine, Department of Pediatrics, Los Angeles, CA, United States of America; 4 Program in Developmental Neuroscience and Neurogenetics, Children’s Hospital Los Angeles, Los Angeles, CA, United States of America; Stellenbosch University, SOUTH AFRICA

## Abstract

Childhood adversity is a potent risk factor for mental health conditions via disruptions to stress response systems. Dysregulations in oxidative stress systems have been associated with both childhood adversity and several psychological disorders (e.g., major depressive disorder) in adult populations. However, few studies have examined associations between childhood adversity, oxidative stress, and mental health in pediatric populations. Childhood adversity (Adverse Childhood Events [ACE]), oxidative stress [F2_t_-isoprostanes (IsoPs)], and mental health pathology were assessed in 50 adolescent females recruited primarily through the Department of Youth Services. Standard ordinary least squares regression models were run co-varying for age, race/ethnicity, adolescent nicotine use, study condition, and parent history of ACEs. Adolescents who reported experiencing four or more ACEs had significantly elevated IsoP levels. Further, internalizing symptom scores across diagnoses were significantly associated with elevated IsoPs, whereas no externalizing symptoms scores, except Attention Deficit Hyperactivity Disorder, were related to altered oxidative stress. Results indicate that IsoPs may be a global marker of childhood adversity and mental health symptomatology, particularly within internalizing symptom domains. A limitation is that body mass index was not collected for this sample. Future studies are needed to replicate and extend these findings in larger, more diverse samples.

## Introduction

Childhood adversity encompasses a range of exposures across infancy through adolescence, such as parental separation or divorce, abuse and neglect, parental substance use problems, family psychiatric illnesses, parental incarceration, and witnessing intimate partner violence. Notably, childhood adversity has been linked to a range of maladaptive outcomes spanning across physical and mental health domains [1] and is a strong predictor of all classes of psychological disorders [2], even after accounting for poor health behaviors [3]. Further, high levels of early life stress and childhood adversity also place individuals at elevated risk of developing physical ailments, such as cardiovascular diseases, obesity, and diabetes mellitus [1, 4, 5]. Mounting evidence suggests that early adversity contributes to maladaptive stress response systems, which in turn, underlie the development of deleterious mental and physical health trajectories throughout the lifespan [1, 6, 7].

### Childhood adversity and mental health

Both retrospective and prospective studies have examined whether childhood adversity is a risk factor for adult-onset psychopathology [8]. Depending upon the specific outcome evaluated, the findings generally indicate that childhood adversity is linked to the development of psychopathology across the lifespan [9], including adolescent-onset [9–12]. A large epidemiological study estimated that childhood adversity is associated with approximately 45% of all childhood-onset disorders and with up to 32% of all later-onset disorders [13]. During adolescence, there is a precipitous rise in several psychiatric illnesses [14], including depression, certain anxiety disorders (e.g., social anxiety disorder, panic disorders), and substance abuse disorder [15]. A review of epidemiological research found that nearly all mental health disorders start by adolescence [16].

Adolescence is an important period of developmental plasticity and sensitivity when brain development is highly dynamic [17]. Significant pubertal, hormonal, neuroendocrine, and metabolic changes occur during adolescence that may interact with early risk factors (e.g., adversity) and contribute to the onset and progression of mental health pathology. Delineating the mechanisms through which childhood adversity confers risk for emerging mental health problems prior to adulthood is imperative to understanding the etiology and progression of psychiatric illness.

### Oxidative stress

Childhood adversity is associated with *both* physical and mental health outcomes, with limited understanding of the specific mediators of these associations [18]. Metabolic and immune systems have been implicated as potential putative mechanistic pathways underpinning the childhood adversity-health relationship [6, 19, 20]. McEwen and colleagues hypothesized that childhood adversity disrupts development by altering homeostasis through a process termed *‘allostatic load’*, which can have targeted effects on mitochondrial function and the production of reactive oxygen species (ROS), as well as systemic effects on brain, immune and metabolic systems [21–23]. Reactive oxygen species refers to a range of molecules and free radicals that derive from molecular oxygen [24]. The majority of ROS are byproducts of cellular metabolism of oxygen that are internally produced by mitochondria, peroxisomes, and cytosolic enzyme systems; however, stress can also externally increase the production of ROS [25]. The dramatic increase in ROS following periods of prolonged stress can be detrimental [26], contributing to redox imbalance which occurs as antioxidant systems are unable to neutralize the effects of the ROS. Altogether, the imbalance between the ROS and antioxidant defenses results in *oxidative stress*. Oxidative stress has emerged as a candidate homeostatic disruption, with the brain particularly susceptible due to high oxygen consumption [27]. Common measures of oxidative stress include ROS, hydrogen peroxide, sphingolipid and glutathione metabolites, and eicosanoids, such as the family of isoprostanes and catalase activity [28]. Elevated isoprostanes, in particular, have been identified as a gold standard measure of oxidative stress, with the advantage revealing acute elevation in response to pathophysiological states, demonstrated maintenance of elevation over time, and normalized when pathophysiology is resolved [29, 30]. While initially applied in studies of pathogenesis in adults [31–33], more recent studies have demonstrated the capacity to measure elevated isoprostanes in early infancy and throughout childhood related to prematurity, diet and diagnosed disorders [34–38]. Thus, measures of elevated oxidative stress are reflected by isoprostane increases across the lifespan, indicating that the field will benefit from greater inclusion of the isoprostane measure in different contexts.

### Childhood adversity and oxidative stress

Few studies have explicitly examined potential relations between childhood adversity and oxidative stress, particularly in pediatric populations. However, preliminary research from both animal and human models suggests that childhood adversity may contribute to oxidative stress. Animal studies have demonstrated that stressful stimuli lead to an increase of oxidative stress in the brain (as reviewed in, [39]). Overall, maternal deprivation paradigms in rodent models have shown an increase in brain lipid peroxidation and other markers of oxidative stress [40].

The literature on oxidative stress and childhood adversity in humans is limited. To date, the majority of these studies have been conducted in adult survivors of early trauma and most studies rely on indirect biomarkers of oxidative stress, such as telomere shortening, which can be induced and accelerated by ROS, and oxidative stress broadly [41, 42]. In general, adults with a history of childhood maltreatment and trauma have shorter telomeres [43–45], with a small-medium effect size [46].

To our knowledge, few studies have explicitly examined oxidative stress and childhood adversity. Epel and colleagues found that adult women with higher perceived life stress had both shorter telomeres and higher oxidative stress compared to the low-stress group, though these findings were not specific to stress during childhood [47]. In a study of children aged 6–14, perinatal complications were associated with elevations in a marker of lipid peroxidation compared to an unexposed group. Further, lipid peroxidation partially mediated the association between perinatal complications and child externalizing problems [48]. Overall, findings support that childhood adversity and oxidative stress are related, but with limited studies and evidence in pediatric populations.

### Mental health and oxidative stress

A rich body of literature has already established key links between oxidative stress and physical health conditions that are often comorbid with mental health illnesses in adolescent populations, including diabetes mellitus [49] and obesity [50]. Several studies have provided evidence of elevated oxidative stress in depressed adults [51]. A meta-analysis of 18 studies found that oxidative stress is increased in adults with depression, although larger-scale studies are needed [52]. There also is evidence for elevated oxidative stress in adults with anxiety symptomatology [53], Attention Deficit Hyperactivity Disorder (ADHD) [54], and schizophrenia [55].

While the majority of such studies have been conducted with adults, one study did find preliminary evidence of high serum uric acid in adolescents with depression compared to a control group, which may indicate redox disruption [56]. A separate study documented a positive relationship between increased oxidative stress and elevated externalizing problem behaviors in a large community sample of 6–13 year old children [57]. A positive association between oxidative stress and mental health problems also was observed in a study of 495 children aged 6–12. Notably, this relationship was moderated by socioeconomic disadvantage, such that the association was only significant in children with more severe socioeconomic disadvantage [58]. Overall, these studies provide evidence to support the hypothesis that oxidative stress is linked to emerging mental health pathology in child and adolescent populations, and that childhood adversities, such as socioeconomic disadvantage, may play a key role in this association.

### Oxidative stress as a pathway linking childhood adversity and mental health

**Fig 1:**
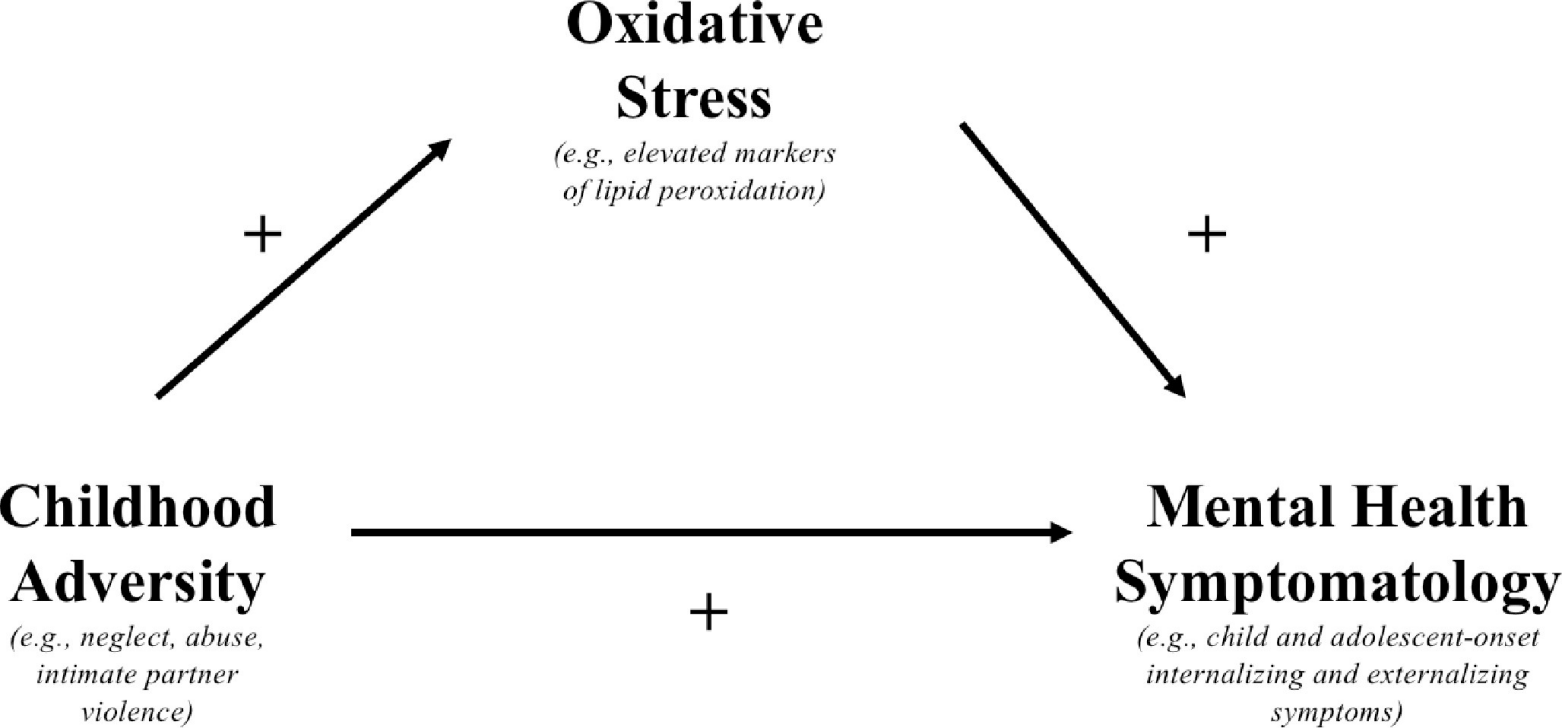
Proposed Mechanistic model of early life adversity, oxidative stress, and mental health symptoms.

Oxidative stress may be a significant contributor to mental health disorders and brain alterations following early life stress, such as child abuse [47]. In adolescence, along with the increase in prevalence of many forms of psychopathology, gender disparities also emerge (as reviewed in, [14]). Most specifically, internalizing disorders are higher in females compared to males [59]. Further, recent evidence has suggested that elevations in oxidative stress for mood disorders, such as depression, are more robust in individuals with early traumatic experiences, particularly childhood sexual abuse [51]. Due to the emerging evidence linking oxidative stress to internalizing psychopathology, and the increase in internalizing pathology in female adolescents, female teenagers represent an important group to investigate for putative relations between childhood adversity, oxidative stress, and mental health. Altogether, while there are a limited number of studies, compelling results suggest that oxidative stress may serve as a mechanistic pathway linking childhood adversity and mental health symptom development. An important future direction will be to examine if oxidative stress mediates the association between childhood adversity and mental health (see Fig 1). A mediation model would indicate that oxidative stress accounts for the association between childhood adversity and mental health problems.

### Current study

In the current study, we aimed to explore the individual associations between childhood adversity, oxidative stress, and mental health in an adolescent sample of girls who were involved in the juvenile-justice system or receiving support services through schools or community agencies. We present results from a supplemental pilot study added to an ongoing intervention trial with 50 adolescent females with a range of early adversity exposure. We first hypothesized that the current study would replicate the common finding that childhood adversity, as indexed by adverse childhood experiences (ACEs) exposure, would be associated with elevated mental health pathology. Next, we sought to characterize a potential association between ACEs and oxidative stress. We hypothesized that ACEs and oxidative stress would be positively associated, such that adolescents with elevated exposure to ACEs would have comparatively higher levels of IsoPs. Next, we examined relative association between IsoPs and a range of mental health symptoms. Given the support for links between oxidative stress and several psychiatric illnesses, we did not have a specific hypothesis regarding differential associations between oxidative stress and certain disorders. Oxidative stress has been linked to a wide range of mental health disorders, including both internalizing disorders (e.g., depression; [51]) and externalizing disorders (e.g., ADHD; [54]). We first examined associations between IsoPs and externalizing and internalizing mental health composite scores. We then tested associations between individual clinical disorders (e.g., depression, ADHD) and IsoPs to explore any possible differential relationships in order to inform future theory development. Optimally, a next step would be to examine a mediation model to examine if and how much IsoPs account for the association between ACEs and mental health. However, due to the pilot and cross-sectional nature of this study, and limited sample size, it was beyond the scope of current study. A power analysis was conducted to confirm the required sample size given an α = .05, 95% power, and a medium effect size, with three predictors. The power analysis determined a minimum sample size of *N* = 63 and confirmed that the current study’s sample size was too small with predicted effects to examine mediation. Thus, this is instead explored as an important future direction with larger sample sizes [60, 61].

## Methods

### Participants

The sample comprised a subgroup of participants from an ongoing randomized control study of an intervention, Safe, Healthy, Adolescent Relationships and Peers (SHARP). SHARP aims to prevent drug use and risk behaviors in adolescent girls. Data in the current study were drawn only from the post-intervention assessment, and therefore detailed information on the intervention can be found elsewhere (Clinicaltrials.gov NCT02420548). The original study included 123 adolescent females aged 13–17 who were assessed both pre and post-intervention after recruitment primarily from the Department of Youth Services (DYS) and through schools and community organizations. For this study, a subsample of 50 adolescents were recruited at the post-intervention assessment. The pilot study was designed during the ongoing study and relevant measures were added following the initial Institutional Review Board (IRB) approval. This project was approved by the University of Oregon Research Compliance Services IRB (IRB Protocol Number: 10312013.040). Written consent for study participation was obtained from parents or legal guardians. Adolescents also provided written assent for participation. The consent procedure was approved by the IRB. Following study initiation, recruitment of the sample was continuous. The subsample did not differ from the larger sample of any key variables of interest (i.e., predictors/outcomes and covariates, *p*>.05).

The self-reported racial and ethnic breakdown of the sample was: 60% non-Hispanic White, 14% multiracial, 8% Black American, and 6% Native American. Further, 20% of individuals identified as Hispanic or Latinx. At the time of assessment, adolescents were between ages 14–19 with a mean age of 16.3 (SD = 1.4). The majority of the sample was recruited through DYS (64%) with the remaining participants recruited through community agencies that provide social services for adolescent girls at-risk for behavioral or emotional difficulties.

### Measures

#### Childhood adversity

At the original study’s baseline assessment, childhood adversity was defined by and indexed through the Adverse Childhood Experiences-Screening Tool (ACE-ST) Scale, a shortened version of the original ACEs measure that assesses 10 possible adverse events occurring prior to the age 18 [1]. These events include: emotional abuse, physical abuse, sexual abuse, emotional neglect, physical neglect, witnessing intimate partner violence, household substance abuse, parental separation or divorce, criminal household member, and mental illness in household. The ACE-ST has been found to demonstrate adequate internal consistency and good construct validity [62]. To preserve confidentiality, participants were instructed to indicate only the *total* number of ACEs they had experienced. The total number of ACEs experienced by respondents was their calculated ACE score. ACEs were examined as both a continuous variable and a categorical variable (dichotomized as Low to Moderate ACE group (0–3 ACEs) and High ACE group (4+ ACEs).

#### Mental health

Children’s mental health symptoms were assessed with the Youth’s Inventory (YI)-4 [63]. The YI-4 is a symptom inventory that contains the behavioral symptoms of 17 emotional and behavioral disorders from the Diagnostic Statistical Manual (DSM-IV). T-scores were calculated for the following disorders: ADHD (Inattention (α = .91), Hyperimpulsive (α = .87), and Combined Type (α = .93), Conduct Disorder (α = .92), Oppositional Defiant Disorder (α = .85), Generalized Anxiety Disorder (GAD) (α = .87), Schizophrenia (α = .74), Depressive Disorder (α = .87), Dysthymic Disorder (α = .85), Bipolar Disorder (α = .69), Anorexia Nervosa (α = .79), Bulimia Nervosa (α = .82), and Substance Use Disorder (α = .86). T-scores were all analyzed as continuous variables.

#### Control variables

The following variables were collected as potential confounders: child age, child race/ethnicity (categorical variable), household income (categorical variable), parent history of ACES (continuous variable), intervention vs control (study condition), and adolescent’s nicotine use (current yes/no).

#### Urine collection and biomarkers assessment

A urine sample was obtained from participants at the post-intervention interview. The sample was aliquoted and stored at a -80°C freezer without preservative. For assessment of oxidative stress, urine was analyzed for levels of F_2_-IsoPs, a stable marker of lipid peroxidation, following previously published protocols [29, 30, 36, 64]. Briefly, 1 ng of [^2^H4]-8-isoPGF_2_α an internal standard, was added to 1ml of thawed urine, followed by F_2_-IsoP extraction using C18 and Sep-pak cartridges. Pentaflurobenzyl esters were generated, purified, converted to trimethylsilyl ether derivatives and then subjected to analysis by tandem gas chromatography/negative ion chemical ionization-mass spectrometry. Urine creatinine was measured to adjust for variation of urine concentration between samples, with measures expressed as ng F2-isoprostanes/mg creatinine (ng/mg Cr).

### Statistical analysis

Descriptive statistics are presented as means and standard deviations. Statistical analyses were performed with SPSS, v. 22 [65].

We first ran zero-order bivariate correlations to examine the following potential confounders: age, household income, parent history of ACES. We ran a one-way ANOVA to examine if there were significant differences in predictors and outcomes for the following categorical variables: nicotine use (Y/N), race and ethnicity, and intervention versus control. All covariates significantly associated with either adolescent ACEs, F2-isoprostanes, or adolescent mental health were included in subsequent analyses.

Three standard ordinary least squares (OLS) regression models were run (Model 1: If ACES predict mental health symptoms, Model 2: If ACEs predict F_2_-IsoPs, Model 3: If F_2_-IsoPs predict mental health symptoms). For the first two models, we first defined a Low to Moderate ACE Group as fewer than four ACE exposures and a High ACE Group as four or more ACE exposures [1]. We also ran the models with the continuous ACE score as a predictor to capture the full range of ACE exposure on mental health symptoms and F_2_-IsoP levels.

For Model 1, a one-way ANOVA was run to test mean difference of mental health T-scores between Low to Moderate and High ACE group. Pearson’s correlations were calculated to assess if the degree to which continuous ACEs were associated with children’s mental health symptoms. If the T-score for a mental health disorder had a non-normal distribution, a Spearman’s rank-order correlation was calculated. In Model 2, we calculated a one-way ANOVA to test if mean F_2_-IsoP levels differed between the Low to Moderate ACE and High ACE Group. A Pearson’s correlation was calculated to test the strength of correlation between continuous ACEs and F_2_-IsoP levels. In Model 3, we assessed the association between F_2_-IsoP concentration and mental health symptoms. We tested correlations between F_2_-IsoP concentration and the T-score of each mental health disorder. We also analyzed whether F_2_-IsoPs were related to composite mental health scores for internalizing disorders (i.e., Anorexia Nervosa, Bulimia Nervosa, Depressive Disorders, Dysthymic Disorder, and GAD) and externalizing disorders (i.e., Conduct Disorder, Oppositional Defiant Disorder, ADHD, Schizophrenia, Substance Use Disorder, and Bipolar Disorder).

Level of significance for all analysis was *p* < .05. However, we also present results with a Bonferroni corrected alpha level of < .004 to adjust for multiple comparisons for a more conservative approach.

## Results

### Clinical and demographic characteristics of the sample

Urine samples were successfully obtained from 50 adolescent females. Clinical and demographic characteristics of the sample are described in Table 1. Slightly less than half of the sample (*n* = 24, 48%) were assigned to the control condition, while the remaining 52% (*n* = 26) of the sample were in the active intervention condition. The number of ACEs endorsed ranged from 0–8, with an average ACE score of 2.69 (SD = 2.26). Thirty percent (*n* = 15) of the sample was in the High Ace Group (4+ ACE Exposures).

**Table 1:** Clinical and demographic characteristics of the sample.

Variable	
Age in years; mean (SD)	16.33(1.40)
Race (*N*, %)	
Caucasian	30(60)
Black American	4(8)
Native American	3(6)
Multiracial	7(14)
Other	6(12)
Ethnicity (*N*, %)	
Non-Hispanic White	33(66)
Hispanic	10(20)
Other	7(14)
Income (*N*, %)+	
Less than $20,000	13(26)
$20,000-$39,999	14(28)
$40,000-$59,999	7(14)
$60,000-$79,999	9(18)
$80,000-$99,999	2(4)
$100,000 or more	3(6)
Child ACE History (*N*, %)	
Low to Moderate (0–3 Exposures)	35(70)
High (4–8 Exposures)	15(30)
F2-Isoprostane Concentration (ng/mg Cr (M, SD)	1.27(.44)
Mental Health T-Scores (M, SD)	
*Internalizing Disorders*	
Anorexia Nervosa	50.10(10.02)
Bulimia Nervosa	47.06(11.19)
Depressive Disorder	50.81(10.33)
Dysthymic Disorder	50.85(11.51)
Generalized Anxiety Disorder	53.31(11.79)
*Externalizing Disorders*	
ADHD-Combined Type	50.49(12.07)
Bipolar Disorder	48.42(9.30)
Conduct Disorder	47.37(7.10)
Oppositional Defiant Disorder	47.04(11.09)
Schizophrenia	49.13(8.75)
Substance Use Disorder	49.76(8.82)

^+^ two participants did not report their income.

### Preliminary covariate analyses

Zero-order correlations between all variables of interest and potential confounders, including age, household income (continuous), parent history of ACE (continuous) were conducted (Table 2). One-way ANOVAs were conducted between all variables of interest and the following potential categorical confounders: race and ethnicity, intervention vs control (study condition), and adolescent nicotine use (current yes/no).

**Table 2:** Zero-Order bivariate correlations.

Variables	1	2	3	4	5	6	7
1. Child Age	-	0.08	-0.06	0.14	0.12	-0.13	0.13
2. Household Income	-	-	-0.05	-0.08	-0.2	-0.22	0.15
3. Parent ACEs	-	-	-	-0.24	-0.14	0.11	-0.16
4. Child ACEs	-	-	-	-	.40**	.44**	.34*
5. Child Internalizing	-	-	-	-	-	.45**	.52***
6. Child Externalizing	-	-	-	-	-	-	0.33
7. Child F2-Isoprostanes	-	-	-	-	-	-	-

* *p* < .05

** *p* < .005

*** *p* < .001

For race, there was no significant association with child ACES (F = 1.18, p = .34), the internalizing composite score (F = .99, p = .46), the externalizing composite score (F = 1.13, p = .37), or F_2_-IsoPs (F = .76, p = .63). For ethnicity, there was no significant association with child ACES (F = .61, p = .62), the internalizing composite score (F = 1.08, p = .37), the externalizing composite score (F = .44, p = .73) or F_2_-IsoPs (F = .19, p = .91). There were no significant associations between race or ethnicity and any mental health disorder T-score (*p>*.10).

There were not significant associations between study condition with child ACES (F = 1.02, *p* = .32), the internalizing composite score (F = .38, *p* = .54), the externalizing composite score (F = .28, *p* = .60), or F_2_-IsoPs (F = 1.02, *p* = .32. There were no significant associations between study condition and any mental health disorder T-score (*p* >.10).

A marginally significant association was observed between nicotine use and child ACEs (F = 3.39, *p* = .05), There was no significant association observed between nicotine use and or F_2_-IsoPs (F = 2.21, *p* = .14). Significant associations were observed between adolescent nicotine use and internalizing composite score (F = 5.54, *p* = .02) and externalizing composite score (F = 24.42, *p* < .001). For internalizing disorders, adolescent nicotine use was significantly correlated with: GAD (F = 5.39, *p* = .03), depressive disorder (F = 5.64, *p* = .022), and dysthymic disorder (F = 4.82, *p* = .03). For externalizing disorders, adolescent nicotine use was significantly correlated with: ADHD-Inattentive Type (F = 6.69, *p* = .01), ADHD-Hyperimpulsive Type (F = 7.41, *p* = .01), ADHD-Combined Type (F = 10.40, *p* = .003), Conduct Disorder (F = 31.64, *p* < .001), Oppositional Defiant Disorder (F = 5.13, *p* = .03), Schizophrenia (F = 6.73, *p* = .01), and Substance Use Disorder (F = 36.10, *p* < .001). Therefore, for models examining the correlation between F_2_-IsoPs and the disorders listed above, adolescent nicotine use was entered as a covariate.

Additionally, a T-score of 70 or higher on the YI-4 indicates a higher degree of clinical severity. For each individual disorder, the proportion of adolescents who endorsed a T-score of 70 or higher can be found in S1 File. It should be noted that the YI-4 is not a diagnostic tool.

### Missing data

There was no missing data for the ACEs or F_2_-isoprostanes. There was a modest amount of missing data for each of the mental health disorders and thus sample sizes dropped due to listwise deletion procedures for relevant analyses. The data were assumed to be missing completely at random. We tested if missingness was related to any of the relevant variables. Missingness was not related to any of the key variables or covariates (*p*>.1). Lastly, all analyses with predictors and outcomes were re-run with mean imputation and results remained consistent.

The sample size for these analyses is as follows: Internalizing Disorders: Composite Score (*n* = 42), GAD (*n* = 49), depressive disorder (*n* = 43), Dysthymic disorder (*n* = 46), Anorexia Nervosa (*n* = 49), and Bulimia Nervosa (*n* = 49). For externalizing Disorders: Composite Score (*n* = 36), ADHD-Inattentive Type (*n* = 44), ADHD-Hyperimpulsive Type (*n* = 45), ADHD-Combined Type (*n* = 43), Conduct Disorder (*n* = 46), Oppositional Defiant Disorder (*n* = 48), Schizophrenia (*n* = 48), Bipolar Disorder (*n* = 49), and Substance Use Disorder (*n* = 46).

Conduct Disorder, Oppositional Defiant Disorder, and Bulimia Nervosa had skewed distributions (kurtosis > 2). Results for analyses including these T-scores are non-parametric Spearman’s rank-order correlations.

### ACEs and mental health

There was a significant difference observed between “Low to Moderate” versus “High “ACE group for mental health symptoms, such that adolescents in the High ACE group were more likely to endorse elevated symptoms from the following disorders: ADHD-Inattentive Type (F = 10.25, *p* = .003), ADHD-Hyperimpulsive Type (F = 4.39, *p* = .042), ADHD-Combined Type (F = 4.57, *p* = .038), GAD (F = 6.11, *p* = .017), Depressive Disorder (F = 8.59, *p* = .01), and Dysthymic Disorder (F = 6.44, *p* = .015). There were no significant correlations observed between ACE group membership and Conduct Disorder, Oppositional Defiant Disorder, Bipolar Disorder, Schizophrenia, Anorexia Nervosa, or Bulimia Nervosa. Further, continuous number of ACEs was significantly correlated with all the disorders listed except GAD (*p*>.05). Continuous number of ACEs was also associated with Substance Use Disorder (*p* < .05). Only ADHD-Inattentive Type was significantly correlated with the High ACE group with the more conservative Bonferroni correction of significance at α < .004.

An internalizing composite score was created for all internalizing disorders (*α =* .89) and externalizing disorders (*α =* .74). High ACE group membership was significantly and positively associated with the composite internalizing score (F = 7.59, *p* = .009) and marginally associated with the composite externalizing score (F = 3.91, *p* = .06).

### ACEs and F2-Isoprostanes

**Fig 2:**
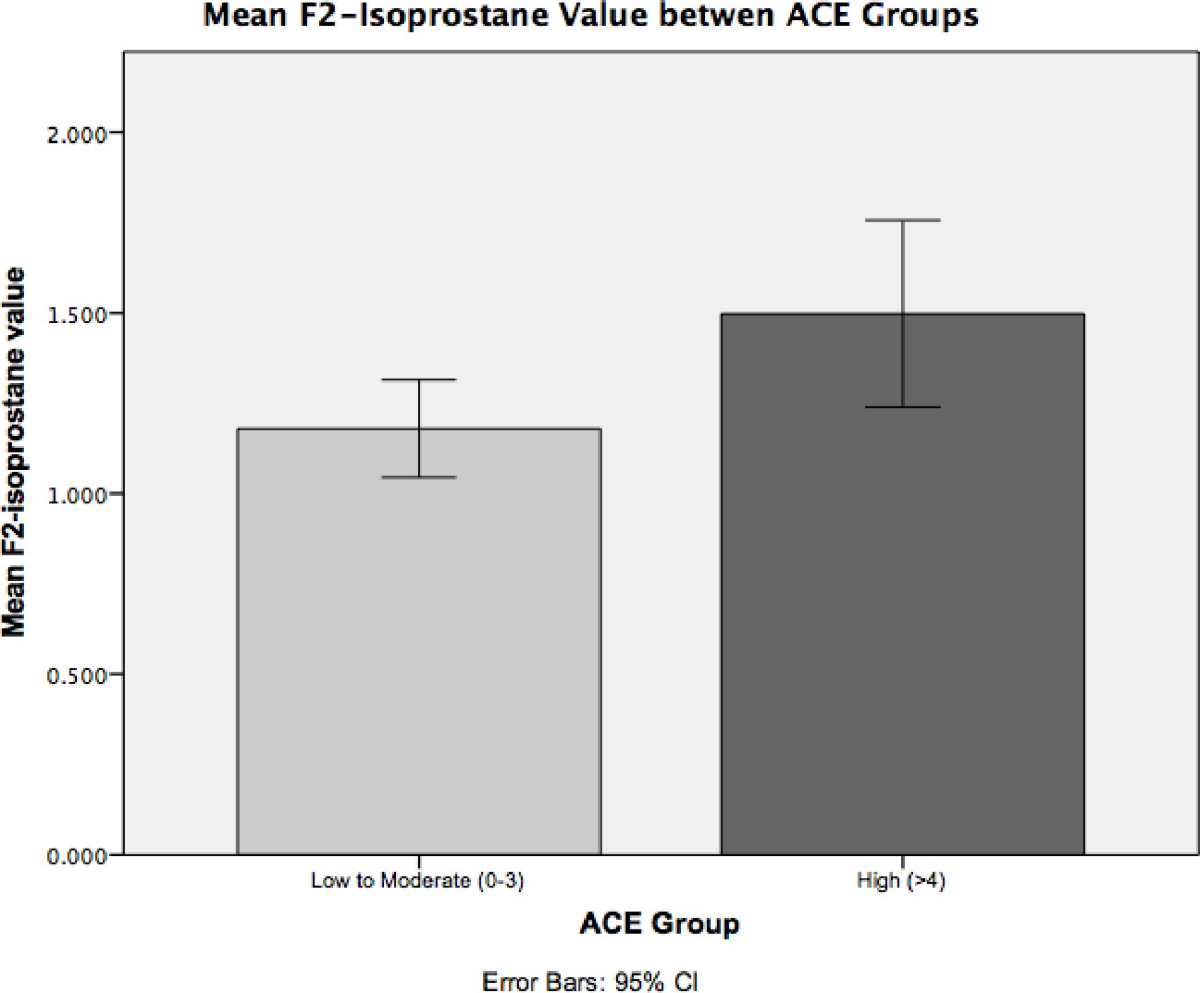
Mean F_2_-Isoprostane Value in Low to Moderate v High ACE Groups.

F_2_-IsoP levels were significantly elevated in the High ACE group compared to the Low to Moderate ACE group (F = 6.13, *p* = .017). F_2_-IsoP levels were .318 ng/mg higher in the High ACE group (*n* = 15) compared to the Low to Moderate ACE group (*n* = 35) (*t* = 2.48, *p* = .017) (see Fig 2). Continuous number of ACE exposures was not significantly related to F_2_-IsoP levels (*r* = .20, *p* = .16).

### F2-Isoprostanes and mental health

Results between F_2_-IsoP levels and mental health symptoms are detailed in Table 3. At α < .05, F_2_-IsoP levels were significantly and positively correlated with all internalizing disorders and the internalizing disorder composite score (*r* = .52, *p* < .001). The correlations between F_2_-IsoP and the following disorders remained significant after statistical adjustment for adolescent nicotine use: GAD (*r* = .37, *p* = .01), Depressive Disorder (*r* = .46, *p* = .003), and Dysthymic Disorder (*r* = .37, *p* = .03). Significant and positive correlations persisted with the conservative Bonferroni correction, and statistical adjustment for nicotine use, between F_2_-IsoP levels and the internalizing composite score (*r* = .50, *p* = .001) and Depressive Disorder (*r* = .46, *p* = .003). The correlation between F_2_-IsoP and GAD remained marginally significant for GAD under the Bonferroni correction of *p* = .004 (*r* = .39, *p* = .005), even when adjusting for nicotine use (*p* = .005).

**Table 3:** Associations between mental health symptom T-scores and F2-isoprostane values.

Disorder	B	SE	β	*t*	*p*
*Internalizing Disorders*					
*Composite Score*^a^	51.81	13.37	0.52	3.88	<0.001^*+*^
Anorexia Nervosa	7.26	3.14	0.32	2.31	0.025
Bulimia Nervosa	8.96	3.47	0.35	2.59	0.013
Depressive Disorder^b^	11.08	3.11	0.49	3.56	.001^*+*^
Dysthymic Disorder^b^	9.49	3.56	0.37	2.66	0.011
Generalized Anxiety Disorder^b^	10.55	3.59	0.39	2.94	0.005
*Externalizing Disorders*					
*Composite Score*^a^	37.11	18.53	0.33	2	0.053
ADHD-C^b^	12.03	3.65	0.46	3.3	.002^*+*^
ADHD-I^b^	10.11	4.23	0.35	2.39	0.022
ADHD-H^b^	10.48	3.41	0.42	3.07	.004^*+*^
Bipolar Disorder	1.41	3.07	0.07	0.46	0.648
Conduct Disorder	1.33	2.35	0.09	0.57	0.574
Oppositional Defiant Disorder	-0.053	3.69	0	-0.02	0.998
Schizophrenia	2	2.89	0.1	0.69	0.494
Substance Use Disorder	5.14	2.88	0.26	1.79	0.081

^a^ Composite scaled score of all internalizing disorders (α *=* .89) and externalizing disorders (α *=* .74)

^b^ Nicotine use was significantly associated with these disorders, and analyses were repeated with nicotine use as a covariate: GAD (*r* = .37, *p* = .01), Depressive Disorder (*r* = .46, *p* = .003), Dysthymic Disorder (*r* = .37, *p* = .03), ADHD-Combined Type; *r* = .41, *p* = .007, ADHD-Inattentive Type (*r =* .31, *p =* .045), ADHD-Hyperimpulsive Type, *r* = .38, *p* = .01).

^*+*^ indicates significant at Bonferroni corrected α = .004

There was a marginally significant correlation observed between F_2_-IsoP levels and the composite externalizing score (*r* = .33, *p* = .053), although this effect was not seen once we statistically adjusted the model for nicotine use (*r* = .30, *p* = .08). In the unadjusted models, F_2_-IsoP levels and ADHD-Combined Type (*r* = .46, *p* = .002) and ADHD-Hyperimpulsive Type (*r* = .42, *p* = .004) were significantly correlated, even following Bonferroni correction. However, when adjusting for nicotine use, the significance diminished, and the correlations were only significant at α < .05 (ADHD-Combined Type; *r* = .41, *p* = .007 and ADHD-Hyperimpulsive Type, *r* = .38, *p* = .01). At α < .05, F_2_-IsoP levels were also significantly correlated with ADHD-Inattentive Type (*r* = .35, *p* = .022), although this also attenuated after adjustment for nicotine use (*r* = .31, *p* = .045).

## Discussion

This study provides preliminary evidence for significant associations between childhood adversity, mental health pathology, and an elevated biomarker measure, F_2_-IsoPs, that may indicate oxidative stress in a female adolescent sample. Thus, the results are the first to demonstrate that exposure to four or more ACEs is linked to potential dysregulation of systemic homeostasis. Further, elevated F_2_-IsoPs were also associated with emerging mental health symptomatology, highlighting that oxidative stress may occur even in subclinical presentations and adolescent expression of psychiatric illness. While it was not a primary aim of this study, we also confirmed that ACE exposure is associated with adolescent internalizing and externalizing problems, a result observed in several prior studies [66–68].

### Childhood adversity and oxidative stress

Results from this pilot study extend the existing literature that childhood adversity is associated with dysregulation of several stress response systems, including metabolic pathways [19]. We demonstrate an explicit link between childhood ACES and oxidative stress in a pediatric population. The initial ACE study found a strong graded relationship between ACE exposure and physical and mental health risk factors and disorders. Specifically, it was noted that persons with four or more categories of childhood exposure exhibited a 4–12 fold increase in risk for smoking, alcoholism, depression, and suicide attempts [1]. Here, we established a similar graded relationship, but between adversity exposure and an elevated biomarker of oxidative stress. Specifically, these results indicated that increased risk (i.e., elevated F_2_-IsoP levels) was observed in individuals who had been exposed to four or more ACEs. Given these findings, further studies are warranted to examine if children exposed to a high degree of childhood adversity are more likely to exhibit oxidative stress across the lifespan.

To date, the majority of this research has been conducted in adult populations with retrospective reports of childhood adversity. However, evidence derived from animal and clinical studies have illustrated that extreme early adversity can “get under the skin” throughout the developmental lifespan [7]. Early adverse experiences contribute to vulnerable phenotypes to psychopathology in childhood via the persistent activation and sensitization of stress-response systems [69]. Dysregulation of metabolic systems during early ages can have persisting, and potentially deleterious, impact on brain structure-function development [7, 70, 71]. The significant elevation of lipid peroxidation in the group exposed to higher childhood adversity events is comparable to findings from animal studies, which have found that early stress exposure (e.g., maternal deprivation) is linked to lipid peroxidation [39, 40, 72]. Prior studies have demonstrated associations between certain forms of early adversity, such as socioeconomic disadvantage [58] and perinatal complications [48], with oxidative stress in human pediatric samples. Increased oxidative stress also has been observed in adult populations with a history of childhood maltreatment [73].

However, we also note that the relationship between continuous ACEs and F_2_-IsoP was non-significant. The significant association between the High ACE group and IsoP was only significant at α = .05, but not following Bonferroni correction. Therefore, results must be interpreted with caution. Additionally, the majority of the sample was exposed to relatively lower levels of childhood adversity (i.e., three or fewer ACEs). Thus, results indicate that in pediatric populations, we may not be able to detect metabolic impacts of lower exposure to childhood adversity. Nonetheless, these findings present preliminary evidence that there may be an important association between childhood adversity and F_2_-IsoPs. Future studies with larger sample sizes, and greater distribution of childhood adversity exposure, will help to establish if this finding is replicable.

### Oxidative stress and mental health symptoms

There is an increasing understanding of the complex relations between brain and peripheral immune and metabolic systems. The challenge remains in elucidating the etiology in mediating the progression of mental health disorders [20]. In adults, there is ample evidence that oxidative stress is observed in individuals with a range of psychiatric illnesses, particularly major depressive disorder [74]. Given the high degree overlap in symptoms and underlying genetic associations between psychiatric illnesses [75], with a renewed emphasis on transdiagnostic associations, it is not surprising that oxidative stress has been reported in other mental health disorders and symptoms [53–55]. Here, we found that higher F_2_-IsoPs was associated with elevated symptoms of all internalizing disorders as well as ADHD. In particular, associations between F_2_-IsoPs and Depressive Disorder were the strongest, remaining significant after a stringent Bonferroni correction and adjustment for nicotine use. We also found a significant association with GAD (*p* = .005), which remained significant at α < .05, and marginally significant with a more conservative Bonferonni correction of α < .004. Recently, in adults, both GAD and depression have been associated with increased lipid peroxidation [76]. Our results indicate that the relationship between oxidative stress with anxiety and depression may also be observed in adolescence and prior to the full onset of the disorder. Lastly, we found a significant correlation between F_2_-IsoPs and ADHD, although this was attenuated following adjustment for nicotine use. Nonetheless, these findings build upon a meta-analysis of six studies reporting that oxidative stress markers were associated with ADHD. Notably, in five of those six studies, the mean age of the population was 9–10 [54].

It should be noted that the mean T-score for all disorders ranged between 48–51. For the YI-4, while it is not a diagnostic tool, a score above 70 indicates a higher degree of clinical severity. It should also be noted that depressive disorder and ADHD (all types) had the highest proportion of adolescent females with a score of 70 or greater (see Supplementary Material). Therefore, it is possible that the strongest associations were observed with these disorders as there were more participants endorsing a greater number of symptoms and severity. Importantly, our results also indicate that F_2_-IsoP concentration is associated with subclinical and emerging levels of internalizing and ADHD psychopathology. An implication of this finding is that F_2_-IsoPs may be a biomarker for the earlier onset of internalizing and ADHD symptoms, which could be useful in detecting children at higher risk for developing the clinically diagnosed disorder. Future prospective longitudinal research is needed to determine whether elevated F_2_-IsoPs predict mental health disorder diagnosis.

The present data do not support an association between oxidative stress and externalizing disorders other than ADHD. Other pediatric studies have found evidence for an association between externalizing symptoms and oxidative stress [57], but no support for this relationship was obtained from the current cohort. It is important to note that the sample in the current study was female, and internalizing symptoms were generally endorsed at higher rates than those in externalizing categories, with the exception of eating disorders. Further, during adolescence, rates of internalizing disorders are greater in females than males [59]. It is possible that in a larger and gender-diverse sample, it would be possible to detect associations between oxidative stress and externalizing symptomatology.

Additional research across development is necessary to characterize the associations between oxidative stress and specific clinical symptoms. For example, an important future direction will be to examine links between transdiagnostic clusters (e.g., sleep disruptions, anhedonia) and oxidative stress. Such research would contribute to a more precise understanding of any differential metabolic functioning between phenotypes. Overall, this would enhance the predictive power of biomarkers, such as F2-isoprostanes, in more effective identification of high-risk children, but also to increase the likelihood of predicting the most likely emergence of specific mental health symptoms.

### Limitations

There are several limitations to the present study. First, there is a limited sample size for this pilot study that was added to an on-going clinical trial. The sample was also non-diverse in terms of gender and relatively less diverse regarding race and ethnicity. Second, there was missing data for the analyses of mental health disorders. Third, this was a moderate risk sample (i.e., 70% of the sample experienced three or fewer ACE exposures) and the majority of the sample reported relatively low levels of mental health pathology (i.e., less than a quarter of the sample endorsed clinically elevated symptom levels, see Supplemental material). However, we did observe robust associations (*r>*.4), particularly between F_2_-IsoPs and Depressive Disorders, ADHD-Hyperimpulsive Type, and GAD. Given the restricted range of T-scores, it is possible that lower correlations were observed than if there were a greater distribution of scores. While F_2_-IsoP concentration was associated with all internalizing disorder symptoms and ADHD, there are weaknesses due to multiple comparisons and elevated risk for Type I error. To mitigate this, Bonferroni correction was applied, and demonstrated a strong association between F_2_-IsoPs and Depressive Disorders, while other relationships were significant at α< .05 (e.g., eating disorders and oxidative stress), suggesting that conclusions should be drawn more carefully.

The study also was unable to collect data on significant confounders that are important in oxidative stress-related research [77]. Of highest concern is the lack of data for body mass index (BMI) or waist circumference. Increased oxidative stress has been associated with obesity in adults, and adiposity can be causally related to the production of ROS [78, 79]. Therefore, we cannot be certain that our findings would not have been modified by adiposity. It should be noted that compared to adults, obesity in childhood may be less chronic and therefore potentially less of a strong confounder with metabolic disruption [80] and psychopathology [81]. Other important confounders include parental mental health and medication use by study subjects. A future direction will be to replicate these findings in a larger study designed specifically to examine these variables.

Additionally, we did not have item-level data on ACE exposures. Therefore, we can only characterize that high ACE exposure broadly was associated with elevated F_2_-IsoPs and mental health. It is possible that certain types of ACE exposure are more heavily associated with disruptions to metabolic functioning. Further, while we did not find evidence of a relationship between continuous number of ACEs with oxidative stress, we note that the High ACE group only contained an *n* = 15, with the majority of the sample having fewer than 4 ACE exposures. A larger sample likely will provide a wider range of adversity exposure to explore more fully the associations between childhood adversity and oxidative stress.

## Conclusions and future directions

Our results, obtained in a pilot study, provide initial support that there are potentially important relations between childhood adversity, oxidative stress, and mental health symptom development. These relationships have been explored more fully in adult samples; the present study provides emerging support that associations can be detected in adolescence. Future research will benefit from extending these results in adolescence and exploring these relations at earlier stages of development (e.g., infancy, middle childhood) using a longitudinal framework.

The study was too small to examine a full mediation model. However, our results provide a foundation for examining whether oxidative stress may mediate the association between childhood adversity and mental health symptomatology. It is also possible that F_2_-IsoPs also moderates this association. Future well-powered studies with longitudinal designs will be able to fully explore whether F_2_-IsoPs mediate or moderate the association between childhood adversity and mental health pathology. It is possible that childhood adversity may only predict mental health symptomatology, or would be a stronger predictor, in individuals with dysregulation of metabolic pathways, such as oxidative stress. If there is a moderation or mediation effect this would support the hypothesis that F_2_-IsoPs, an index of lipid peroxidation and oxidative stress, is a reliable biomarker of toxic stress to help identify children at highest risk. This will have strong implications for prevention and intervention work.

The potential outcomes of childhood adversity are severe. We urgently need innovative strategies to delineate *how* and *why* adversity impacts health so broadly, to understand developmental trajectories, and to recognize the children in most need of support. Taken together, this knowledge can be leveraged to alleviate the consequences of childhood adversity and toxic stress.

## Supporting information

S1 File Proportion of adolescent females with a T-score of 70 or greater on Youth’s Inventory. (XLSX)

S1 Material (DOCX)
