# Supplementary material for: Childhood adversity, mental health, and oxidative stress: A pilot study
Source: PLoS One. 2019 Apr 26;14(4):e0215085. doi: 10.1371/journal.pone.0215085 (PMC6485615; doi:10.1371/journal.pone.0215085)
Supplement: S1 Material — (DOCX) [file pone.0215085.s002.docx]

Supplementary Material:

The proportion of adolescent females who endorsed a T-score of 70 or higher on the YI-4 (indicating a greater degree of clinical severity) for each disorder is presented below:

For internalizing disorders, number and percentage of adolescent females who endorsed a T-score of ≥70*:* GAD (*n*=7, 13.7%), Depressive Disorder (*n*=10, 19.6%), Dysthymic Disorder (*n*=8, 15.7%), Anorexia Nervosa (*n*=4, 7.8%), and Bulimia Nervosa (*n*=6, 11.8%).

For externalizing disorders, number and percentage of adolescent females who endorsed a T-score of ≥70: ADHD-Inattentive Type (*n*=11, 21.6%), ADHD-Hyperimpulsive Type (*n*=10, 19.6%), ADHD-Combined Type (*n*=12, 23.5%), Conduct Disorder (*n*=6, 11.8%), Oppositional Defiant Disorder (*n*=5, 9.8%), Schizophrenia (*n*=5, 9.8%), Bipolar Disorder (*n*=3, 5.9%), and Substance Use Disorder (*n*=6, 11.8%).
